# Recovery of Rare Earth Elements by Carbon-Based Nanomaterials—A Review

**DOI:** 10.3390/nano9060814

**Published:** 2019-05-29

**Authors:** Celso E. D. Cardoso, Joana C. Almeida, Cláudia B. Lopes, Tito Trindade, Carlos Vale, Eduarda Pereira

**Affiliations:** 1 Chemistry Department, CICECO and CESAM & LAQV-REQUIMTE, University of Aveiro, Campus de Santiago, 3810-193 Aveiro, Portugal; cedc@ua.pt (C.E.D.C.); joana.cruz.almeida@ua.pt (J.C.A.); claudia.b.lopes@ua.pt (C.B.L.); tito@ua.pt (T.T.); 2 Interdisciplinar Centre of Marine and Environmental Research, 4450-208 Matosinhos, Portugal; carlos.vale@ciimar.up.pt

**Keywords:** carbon nanostructures, E-waste, rare earth elements, solid phase extraction, sorption

## Abstract

Modern societies depend strongly on electronic and electric equipment (EEE) which has a side effect result on the large production of electronic wastes (e-waste). This has been regarded as a worldwide issue, because of its environmental impact—namely due to non-adequate treatment and storage limitations. In particular, EEE is dependent on the availability of rare earth elements (REEs), considered as the “vitamins” of modern industry, due to their crucial role in the development of new cutting-edge technologies. High demand and limited resources of REEs in Europe, combined with potential environmental problems, enforce the development of innovative low-cost techniques and materials to recover these elements from e-waste and wastewaters. In this context, sorption methods have shown advantages to pre-concentrate REEs from wastewaters and several studies have reported the use of diverse nanomaterials for these purposes, although mostly describing the sorption of REEs from synthetic and mono-elemental solutions at unrealistic metal concentrations. This review is a one-stop-reference by bringing together recent research works in the scope of the application of carbon nanomaterials for the recovery of REEs from water.

## 1. Introduction

Emerging key technologies related namely to renewable energy, energy efficiency, electronics, and aerospace industries, have an important role in the current socio-economic. Environmental and public health risks are still poorly documented. Pursuing solutions to support the transition towards a sustainable lifestyle is thus crucial. In this context, the increasing use of raw material containing the designated technology-critical elements (TCEs) and the environmental impacts derived from mining to end-of-life waste products are of common concern. European Union proposed this label for the elements because there are no mining zones with an acceptable short/mid-term profit of these elements within the EU borders [1]. Activities related to the availability of those elements are hence of economic relevance. TCEs include Ga, Ge, In, Te, Nb, Ta, Tl, the platinum group elements (PGEs: Pt, Pd, Rh, Os, Ir, Ru), and most of the rare earth elements (REEs: Y, La, Ce, Pr, Nd, Sm, Eu, Gd, Tb, Dy, Ho, Er, Yb, Lu). 

Currently, the extraction of TCEs from ores involves large energy costs and has potential environmental risks because of the chemicals used during mining and extraction operations [2]. An alternative source to obtain TCEs is from recycling waste electrical and electronic equipment (e-waste). However, recycling is generally expensive and shows low efficacy [3,4,5,6,7,8,9,10,11,12]. Although the use of TCEs is in the infancy, recent studies have reported enhanced concentrations of various REEs in waters of the Rhine River (Germany) [13] and of the San Francisco Bay (USA) [14]. TCEs can be released into the environment and become in contact with any stage of the life cycle [15]. Searching more effective technologies that respond to manifold challenges in the recovery of TCEs from Waste Electrical and Electronic Equipment (WEEE) is, therefore, an emerging and pertinent issue.

## 2. Waste of Electrical and Electronic Equipment

**Figure 1:**
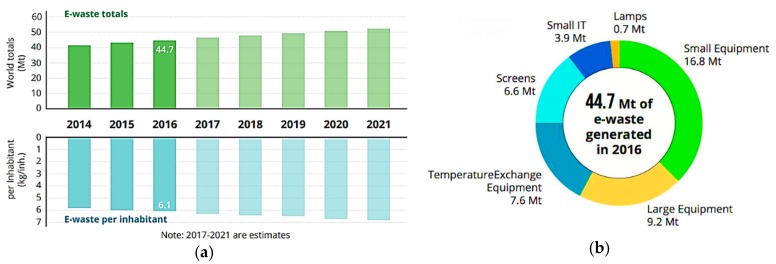
(**a**) Amount of global e-waste generated from 2014 to 2016 and values estimated for the following years (2017 to 2021) and (**b**) typology of e-waste produced in 2016. Adapted from Baldé et al. (2017) [18], with permission from ITU, 2017.

There are several definitions of e-waste. In this work e-waste is considered a specific form of waste that mainly covers old, end-of-life and/or discarded appliances that used electricity for working. Examples of e-wastes are highly consumed electronics (e.g., computers, Liquid-Crystal Display (LCD) screens, smartphones), large appliances (e.g., refrigerators, washers or dryers) and other similarly consumed products that are discarded by their original users or by having a manufacturing defect [16,17]. The reported quantity of e-waste generated worldwide in 2016 was 44.7 million tonnes (Mt), which correspond to 6.1 kg per inhabitant, and is expected to reach 52.2 million tonnes in 2021 (Figure 1).

The economic value of raw materials containing e-waste was estimated as 55 Billion Euros in 2016 [18], and REEs recycling market in Europe is estimated to be worth 1 billion euros [19]. Although recycling has been strongly encouraged only 9% of e-waste has been collected [19], and the recycling rate of REEs is less than 1% worldwide. A number of reasons contributed to this low value, such as the proliferation of EEE in consuming modern societies, the relatively short lifetime of products, and the complexity in TCEs recycling and recovery processes. Increase in research and development aiming at the REEs recovery technologies had little impact on the industrial sector with the exception of the recovery of REEs from lamp phosphors [17,19].

## 3. Technology-Critical Elements

**Figure 2:**
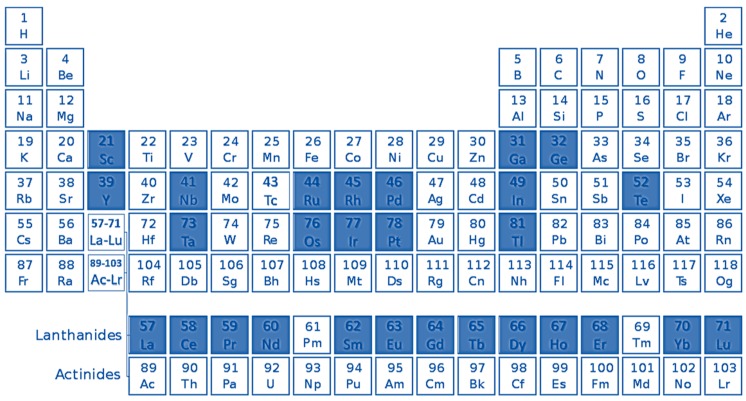
Periodic Table of the Chemical Elements showing in full blue squares the technology-critical elements (TCEs).

Supply and demand of TCEs in high-technology, energy supply and green applications are unbalanced. Figure 2 shows in red Ga, Ge, In, Te, Nb, Ta, Tl, the platinum group elements (PGEs: Pt, Pd, Rh, Os, Ir, Ru), and most of the rare-earth elements (REEs: Y, La, Ce, Pr, Nd, Sm, Eu, Gd, Tb, Dy, Ho, Er, Yb, Lu) [20]. This review focus on the recovery of REE and, therefore, a brief introduction to such elements is presented below, with emphasis on the lanthanide series.

### 3.1. Rare-Earth Elements: Properties and Applications

**Figure 3:**
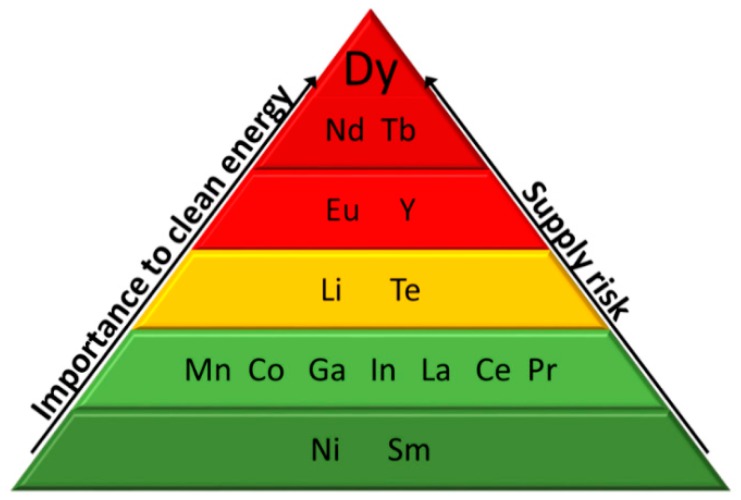
Criticality assessment of rare earth elements (REEs) and other elements in the medium term (2015–2025). It is represented in green the elements that are not critical, in yellow the near-critical elements and in red the critical elements.

**Figure 4:**
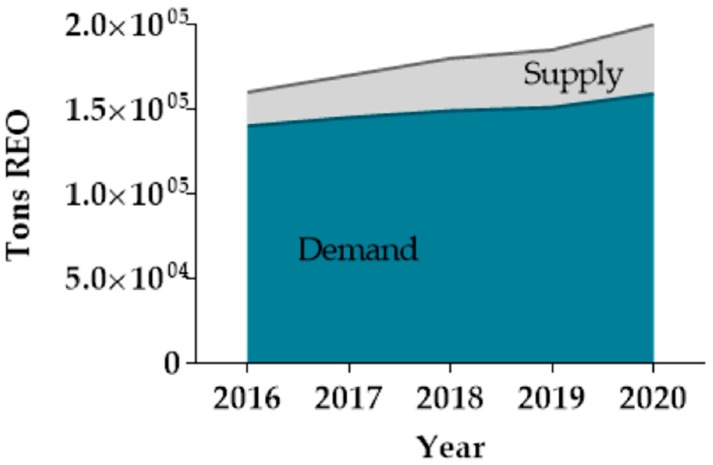
Evolution of global REE demand and supply from 2016 to 2020. Data obtained from [24].

The International Union of Pure and Applied Chemistry (IUPAC) defines the REEs as a group of 17 elements comprising the 15 lanthanoids (La to Lu) plus scandium (Sc) and yttrium (Y). These two elements tend to occur in the same ore deposits as the lanthanoids and to share many chemical and physical properties [19]. REEs are usually subdivided into light REEs (LREEs), heavy REEs (HREEs) and middle REEs (MREEs), although this classification has not been consistently adopted in the literature [21]. In general, LREEs includes the elements from La to Pm, MREEs are Sm, Eu and Gd, and the elements between Tb and Lu are designated as HREEs. Yttrium is included in this group, due to a similar ionic radius and chemical properties. Scandium does not share enough similarities to be classified in any of those classes. Most of REEs occur together in mineral deposits often widely dispersed and at very low concentrations (10 to a few hundred ppm by weight). Ore mining is energy intensive process and generates large quantities of waste [22]. For example, production of 1 tonne of rare earth oxide (REO) in China generate 60,000 m^3^ of waste gases, 200 m^3^ of acidified water and 1.4 tonnes of radioactive waste since of most REEs deposits contain uranium or thorium. In addition, high energy is required, generally derived from conventional sources [23]. Figure 3 illustrates Dy, Tb, Y, Eu, and Nd [24] as critically at-risk marketable elements. Figure 4 represents the evolution of global rare earth oxides demand and supply from 2016 to 2020 [24]. 

In order to provide the expected increasing demand, new projects may occur for exploring and mining ores containing REEs. These elements usually are present at oxide, silicate and phosphate minerals, such as apatite, monazite or xenotime, bastnaesite and perovskites. Monazite and bastnaesite are the main minerals of LREEs and xenotime is the main source for HREEs namely Y, Dy, Ho, Er, Tm, Yb and Lu [21].

The luminescence of Lns and their compounds has been applied for a number of technological applications, that results from electronic transitions occurring within the f shell, which is well shielded inside the atom and less sensitive to the local environment. As such, for each Ln, the luminescence spectra appear with characteristic sharp bands sharing strong similarities with the respective spectrum of the free ion. Furthermore, the luminescence lifetimes of lanthanoids are typically within the millisecond time-scale, which is superior to those observed for organic fluorophores, which might be useful for time-gate monitoring applications [25,26]. The luminescence of Ln^3+^ can be used in a wide variety of practical applications also because it covers a large spectral window depending on the Ln used, from the UV (Gd^3+^), through the visible blue (Tm^3+^), green (Tb^3+^), orange (Sm^3+^) and red (Eu^3+^), to the near infrared (NIR) (Pr^3+^, Nd^3+^, Er^3+^ and Yb^3+^) [25,26].

### 3.2. Industrial Applications of Rare Earth Elements

Table 1 and Table 2 list the application of REEs in industrial domains. In the category of LREEs, La is mainly used in fluid cracking catalysts (FCC) and batteries; it is an important element in hybrid car batteries, since acts as a hydrogen absorber in rechargeable batteries [22]; the main application of Ce is in polishing, although Ce is also used in catalytic converters for automobiles (up to 30%), glass and metallurgy, and shows a strong affinity for elements like phosphorus, making them suitable as water purifiers; Pr is used in magnets and phosphors; and the Nd highest application are in magnets. In addition, both La and Ce act as stabilizers in catalytic compounds, such as in oil refineries. In the category of MREEs, Gd has a wide range of applications that include applications in magnets, metallurgy and phosphors industries; Sm is widely used in magnets and Eu in phosphors because of its excellent luminescent pigment properties. Finally, in the category of HREEs, Tb is used in magnets and phosphors, Er is applied in phosphor and glass industry, and the Y is used in ceramics and phosphors. Although generally used in small quantities, they are nonetheless essential elements in such functional devices and the technological demand for these elements will tend to increase in the future. [20,22].

**Figure 5:**
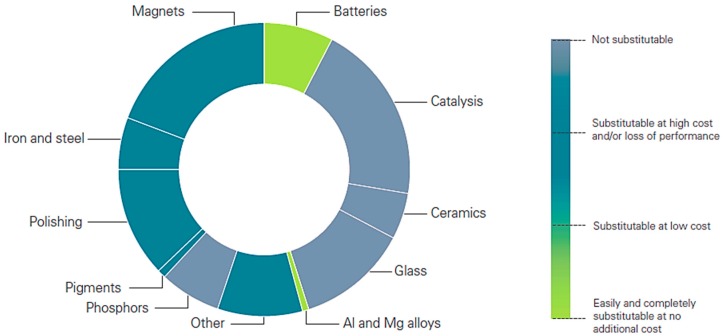
Current consumption of REEs in several applications, as well as the respective susceptibility to be replaced [22].

Among the variety of REEs applications, there are four main target markets—magnets, metallurgy, catalysts and polishing powder—which account for nearly three quarters of the total use of rare earth elements in 2012. Other important applications are as pigments in glasses, phosphors and ceramics [22]. Figure 5 shows the current consumption of REEs in several applications, as well as the respective susceptibility of being replaced. REEs are particularly relevant in catalysis, phosphors, ceramics and glass industry, and difficult to replace in the case of magnets, iron and steel applications.

General description of the principal applications of REEs [20,27,38] is as follows:

- **Magnets**: Many REEs have important magnetic applications, such is the case of Neodymium-Iron-Boron magnets, which also contain Pr, Dy, and Sm ions. The main applications for permanent magnets are industrial motors, hard-disc drives, hybrid and electric vehicles, and wind turbines.
- **Batteries**: Nickel metal hydride batteries (NiMH) are excellent for portable electronics, containing mainly La and Ce ions. NiMH batteries have also been extensively used in hybrid and electric vehicles; however, its dependence is decreasing with the growing and development of more efficient Li-ion batteries.
- **Metallurgy**: Light rare earth are used to improve the mechanical characteristics of alloyed steel, for desulfurization, to bind trace elements in stainless steel and in magnesium and aluminum alloys.
- **Catalysts**: REEs have an important role in catalysis, for example, La used in fluid catalytic cracking (FCC) in oil refineries—which increases oil refinery yields by up to 7%—, and Ce in catalytic converters for cars.
- **Polishing powder and glass additives**: Cerium oxide is widely used as a polishing agent and as an additive in the production of glass, in the discoloration and removal of impurities.
- **Phosphors**: End-of-life fluorescent lamps are a rich source of heavy rare earth elements (HREEs), such as Eu, Tb and Y. HREEs are important constituents of tri-phase phosphor lighting used for linear fluorescent lamp tubes and compact fluorescent lamps, as well as LCD backlights for flat panel displays.

### 3.3. Evidence of Anthropogenic Rare Earth in Aquatic Systems

REEs in non-recycled waste materials may end up in surface waters and ocean. For example, the highest REEs concentration in water wells in Chinese mining areas was reported as 130 μg/L; however, Sidaosha River—which has the highest level of REEs—has a total of REEs concentration in suspended particles and surface sediments of 31,524 and 30,461 μg/g, respectively [39]. Recently, high concentrations of rare earth have been detected in the surface water of San Francisco Bay [14], presumably, due to the wastewater treatment plant discharges of refractory magnetic resonance imaging (MRI) contrast agents used in hospitals and medical research centres [24]. In addition, it has been found that anthropogenic Gd contaminates surface and ground water, which highlights the need for wastewaters treatment. Indeed, wastewater can also be a source of REEs although their potential remains largely unexplored, as is the case of wastewaters produced during the extraction and separation of the elements. However, the recovery of REEs from acidic industrial waste water streams and mining effluents is still in its infancy, since their concentrations in industrial waste residues are very low compared to primary rare earth ores, whereby it is necessary develop special processes dedicated to the recovery of rare earth from these dilute waste streams.

## 4. Recovery of Rare Earth from E-Waste

### 4.1. Recycling and Barriers to End-of-Life Recycling

The major constraint in recycling REEs has been the low yield rate, which can be explained by the lack of adequate recycling design and by the number of steps required for their separation. These limitations have contributed to the false premise that recycling is not profitable, due to the small quantities recovered [28]. Nevertheless, several examples of REEs recovery from end-of-life products have been presented, such as from fluorescent lamps [27,29,36,37], magnets [30,31,32,33,34], NiMH batteries [40] and mobile phones [41]. Many of these studies report yields up to 99% of re-usable REEs. This scenario is illustrated by the study of Kim et al. [42] on the recovery of REEs oxides (namely Nd, Pr and Dy) from commercial NdFeB magnets and industrial scrap magnets by employing membrane-assisted solvent extraction and without any co-extraction of non-REEs over the 120 h run. Although many lab-scale experiments have reported good REEs recovery, recycling techniques in an industrial context are still in its infancy [43]. However, recently, some chemical and electrical companies are cooperating with each other, in order to develop techniques and processes to recover high purity REEs from e-waste [24]. A mature recycling route for REEs could offer a number of important advantages over primary production, such as a smaller environmental footprint (even because recycling does not leave radioactive elements to dispose of), shorter lead times and a cheaper source of material compared to primary production.

Many of the recycling techniques which are applied to the e-waste and end-of-life products, including liquid-liquid extraction processes or the use of sorbents for solid-phase extraction, either in batch or column approaches, are common for different types of products. Conversely, there are products that might be specifically treated, such as metal alloys in magnets [32,40]. It is also important to create recycling policies and networks for these products so that they can also compete—in terms of price and quality—with rare earth materials produced from primary sources. This can be accomplished by increasing the cost-efficiency and the competitiveness of dismantling, sorting, separating and re-processing of end-of-life products containing REEs and by improving the yield of various REEs recovery routes. Furthermore, strategies for preserving primary REE resources should be also implemented [28]. Regardless the recycling techniques used, several barriers need to be surpassed in order to implement recycling of REEs containing products at a large scale [22], such as: (i) Insufficient and often non-selective collection rates; (ii) Lack of information about the quantity of REE materials available for recycling; (iii) Dissipative use, since the quantity of rare earth per component or device is often very small, which can make it difficult to detect the REEs products in mixed waste streams and uneconomical to separate them; (iv) Presence of contaminants; (v) Price volatility for scrap and products like magnets or phosphors; (vi) Shipping of e-waste.

### 4.2. Steps of e-Waste Treatment

**Figure 6:**
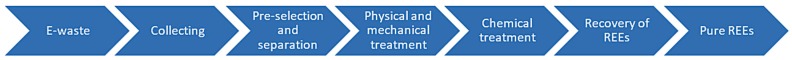
Steps of a general process of REEs recycling from e-waste.

The different typology of the products containing REEs offers different challenges in the process of recovery of REEs. In a first step it is necessary that the e-waste undergo mechanical or physical treatments. At this stage, the e-waste is dismantled, separated and crushed by mechanical (shredding, cutting, grinding or milling) and by physical processes (separation by vibration, gravity, buoyancy, magnetic or Eddy current) [44]. The next step is a chemical decomposition by leaching or chemical treatment for later recovery of REEs from aqueous systems (Figure 6) [45].

It is important to note that the rare earth compounds presented in the products are intricately embedded into them—as exemplified by neodymium magnets from hard disks and compressors, electronic displays—, and are of different nature; therefore, it is recycling from consumer goods at end-of-life is usually more challenging. In this way, the recycling process needs efficient and effective physical and chemical separation techniques to be effective [46].

It is possible to observe in the literature that before starting the e-waste recycling process (namely, the recovery of the elements) there has been a pre-selection and separation of the different types of e-waste. This approach is justified by the nature, typology and constitution of e-waste that is very different from each other, whereby it offers different challenges (for example, in dismantling and treatment of e-waste) and requires more specific methods depending on the type of e-waste which it is dealing with. Even because, a common approach to e-waste treatment would become infeasible because of the way in which electronic products are produced and to the different constituents of the products. In this way it is recommended to create a “recycling line” depending on the type of end-of-life products dealing with. However, in the case of effluents, it becomes even more difficult to recover elements from the water since it has a more diversified composition. A solution might be the use of materials that are not very selective between the different rare earth, but which are between rare earth and the other elements. However, the mixture of effluents with very diverse nature could become the recovery of these elements impracticable, due to a higher entropy in the system by the elements, making the matrix too complex. 

### 4.3. Overview of Recovery Methodologies to Aqueous Systems

Liquid-liquid extraction (LLE) and solid phase extraction (SPE) approaches have been applied to achieve a viable method for the separation and extraction of REEs. LLE implies two immiscible liquids (aqueous and organic solvents) to separate compounds through the attraction of the desired element from one side of the liquid phase towards another liquid phase [47]. Among the large-scale LLE techniques, there are metallurgical processes where metals are melted by heat—pyrometallurgical processes—or dissolved by a liquid—hydrometallurgical processes [45]. Pyrometallurgical techniques have commonly been used in the last three decades, due to the advantage of melting any forms of scrap [24,47]. Disadvantages are the generationally large amount of slag, loss of precious metals, and difficulty in recovery some metals [27,45]. Hydrometallurgical techniques include leaching, ion exchange, solvent extraction, and precipitation that conducts separation and extraction of metals based on the reaction in an aqueous medium. Leaching solvents commonly used are H_2_SO_4_-H_2_O_2_, aqua regia, thiourea, cyanide, HNO_3_, NaOH, HCl, etc. [27,45,47]. Hydrometallurgical techniques are easier to control and create less environmental hazards than the pyrometallurgical approach. However, high operating temperature and high consumption of concentrated chemicals produce a large amount of liquid wastes and toxic sludges [24,47]. Table 3 reports a few examples of studies on hydrometallurgical methods to recovery REES, as well as the references Dutta et al. [24], Kaya [45] and Hidayah and Abidin [47].

SPE is an extraction process of the desired element from the liquid phase towards the solid phase [47]. Among the different separation and preconcentration techniques, batch and column approaches are those that have been widely used [38]. Although LLE was more used at industrial scale, its efficiency was always questionable because of the inability to extract polar compounds, tendency to form emulsions, presence of impurities in final product, loss of extractant into aqueous phase, laborious and time-consuming, low purity of the products, and disposal of toxic or flammable chemicals [47]. In this sense, SPE offers the advantages of having a large surface area and better contact between extractant and REEs in the aqueous phase, which led to an increase of the extraction efficiency, selectivity, quality, contact area, and reduce chemical consumption in the separation and extraction of REEs [57,58].

#### 4.3.1. Preconcentration Methods—Solid-Phase Extraction

In general, a preconcentration method should be fully validated, efficient, rapid, use a low volume, or ideally, no harmful solvents neither require a large volume of sample [59]. When compared to LLE, the solid phase extraction offers a number of important advantages, such as reduced organic solvents usage and exposure, high enrichment factor, rapid phase separation and the possibility of combination with different detection techniques [38].

There are two basic approaches that can be recognized in SPE, namely column (or online) and batch (or offline) procedures. In the column approach, operations run out automatically, leading to a high sample throughput with lower prone to airborne sample contamination. Automation enables the analyst to undertake other tasks during the analysis of the samples and decreases the possibility of human error during the preparation; moreover, the conditions obtained are reproductible. It should be noted that a high enhancement factor does not represent an effective preconcentration. Although preconcentration efficiency depends on the time that the sample passes through the column, the enrichment can be improved by the use of a longer loading time or a higher flow rate. However, this leads to a decrease in the sampling rate [38,59]. In batch preconcentration procedures, the retention medium is mixed with the liquid sample, then filtered, and finally, the enriched phase is transferred to the detector. All these operations occur manually. Several nanomaterials have been investigated as solid phase sorbents in batch technique, such as polymers supports, carbon-based composites (carbon nanotubes and graphene oxide) and nanoparticles (NPs) in particular those exhibiting magnetic properties [60,61,62]. 

**Ion imprinted polymers.** Ion imprinted polymers (IIPs) are highly crosslinked polymers, which have specific binding sites for a particular metal ion. The process consists in making a template (the print metal ion on its complex) by coupling chemically with one or several functional monomers and then spatially attaching in a solid polymer by the polymerization reaction. After the imprint ion removal, the polymer with the template is obtained. This structure is complementary—in size, shape and functionality—to the ion removed, whereby it will have high selectivity to the ion. This approach is a big advantage in very specific applications that require extremely selectivity to separate elements with very similar chemistry. The interactions between the polymer framework and the complexed ion are based on coordinative bonds from some electron donating heteroatoms (such as oxygen, nitrogen or sulphur) to the unfilled orbitals of the outer sphere of the metal ions. IIPs are mainly prepared by free radical polymerization where vinyl groups are the classic type of polymerizable functions. The used ligands act as bifunctional reagents and their functionality comes both from the chelating ability and the vinyl function [38]. An alternative method for IIPs preparation is by trapping of a non-functionalized ligand inside the polymer network; these different ways of incorporating the ligands have been presented in detail in recent reviews [63,64].

**Silica-based supports.** Silica-based materials are good sorbents, due to their porous structure, mechanical properties, physical and chemical stability, convenient preparation, high thermal stability, high acidic stability, simple chemical synthesis, and the possibility to immobilized various functional groups on its surface to enhance the sorption to metal ions. Silica has some drawbacks, such as easy degradation at high pH values and difficulties to separate the sorbents from water under continuous industrial operation [38,47,59].

**Membrane supports.** Membrane supports as polytetrafluoroethylene, polyviylidene fluoride, polyamide, and ceramic membranes are common supporting material used in solid phase extraction of REEs. The main advantage of using membranes in the extraction process is the low consumption of energy, high selectivity on REEs, low cost operation, and the easy to manage [38,47,59].

**Microorganism materials**. Microorganisms, such as *Bacillus subtilis*, *Escherichia coli*, *Pseudomonas fluorescens*, *Paracoccus denitrificans*, *Schwanella putrefaciens*, and *Alcaligenes faecalis* are efficient and environmentally friendly sorbents in the extraction of metal ions. Another advantage of its use is the low use of expensive and toxic reagents. The microorganisms can interact with metal ions through surface adsorption, adsorption on extracellular biopolymer, biologic absorption, and adsorption on extracellular bio-mineral; however, surface adsorption is the more effective process in the REEs extraction [38,47,59].

#### 4.3.2. Sorption a Promising Process to Recover Rare Earth Elements

As already mentioned, various materials can be used as sorbents for the REEs recovery. Also, sorption has many other advantages when compared to the most common techniques, namely high removal efficiency, easy to operate and install, and involve low maintenance costs. Nevertheless, the efficiency of the whole process is dependent on factors that may influence the REEs recovery, either the kinetics or the sorption capacity. These factors are the typology of the sorbent and the metal ion to be recovered, as well as the experimental parameters, such as:

- pH of the batch, because it will affect the metal ions and the sorbent; the surface charge of the sorbents depends on the acidity of the surrounding electrolyte; since the sorption of REEs occur mainly by electrostatic forces, the surface charge of the sorbents needs to be negative;
- Temperature, because inadequate temperatures can decrease the efficiency of the sorption process;
- Dose of sorbent, since theoretically recovery rate increases with the dose;
- REE initial concentration because, for the same dose of sorbent, higher values of concentration, lead to lower sorption rates;
- Stirring speed, which controls the dispersion of particles and the mass transfer rate.

Besides these, the presence of other metal ion species, which is the realistic condition in real effluents, can influence the recovery of REEs due to the competition for the binding site. Therefore, these factors should be considered for the efficient recovery of REEs. Further discussion of this issue is in the Section 4.3.3—A3, B2 and C1.

#### 4.3.3. Carbon-Based Nanomaterials Applied in Solid Phase Extraction

**Figure 7:**
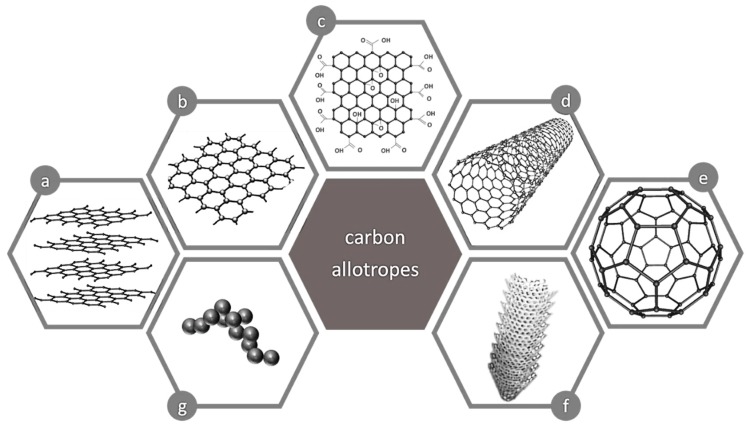
Carbon allotropic forms: (**a**) Graphite, (**b**) graphene, (**c**) graphene oxide, (**d**) carbon nanotube, (**e**) fullerene, (**f**) carbon nanofibers, (**g**) carbon dot.

Recently, increasing attention has been given to carbon nanostructure materials applied in solid phase extraction. Carbon is the essential building block in many of the compounds and materials, due to its capability of having several oxidation states and/or coordination numbers. This makes carbon one of the few elements to have multiple numbers of allotropic forms like graphite, graphene, graphene oxide, carbon nanotubes, carbon nanofibers, carbon dots, among others (Figure 7). The structures of their surface are highly complex and depend on the raw materials, as well as on the production method and pre-treatment process. The sorption of REEs is mainly controlled by electrostatic forces, which are related to the various surface functional groups (O donors). Furthermore, the oxidation of the carbon surfaces provides a more hydrophilic surface and a larger number of oxygen-containing functional groups, such as hydroxyl, carbonyl and carboxyl groups. This variety increases the possibility of further modification and functionalization of the graphitic surface [38].

##### A. Graphene and Graphene Oxide

Graphene is a two-dimensional material composed of covalently linked sp^2^ hybridized C atoms and that has gained high attention, due to its properties, such as high electronic conductivity, good thermal stability, excellent mechanical strength and mainly large surface area (for analytical applications). A significant advantage of graphene-based materials over carbon nanotubes (CNTs) is that for certain applications can be produced from graphite, which is a common and cheap material, without using metal catalysts. [65,66]. Nonetheless, other preparative methods are required for high quality applications, such as in electronics.

Graphene oxide (GO), mainly composed of carbon, oxygen, and hydrogen atoms, is a graphene-based material whose structure differs from the graphene structure in their regions with aliphatic six-membered rings containing hydroxyl, epoxide, carbonyl and carboxyl groups (beyond the aromatic regions with unoxidized benzene rings). Additionally, these oxygen-containing functional groups provide important nucleation sites for further chemical modification, such as decoration and functionalization. Those possibilities make GO a good candidate for usage in polymer composites, energy-related materials and sensors. GO has a high potential for removal of metal ions, due to its extremely hydrophilic properties and the presence of functional groups containing oxygen atoms. However, in water GO surface changes resulting often in an agglomeration and precipitation, due to the neutralization of negatively charged functional groups and the creation of GO–metal complexes [67,68].

A1–Production of Graphene and Graphene Oxide

Graphene can be synthesized by two approaches: bottom-up and top-down. The bottom-up approach consists of conventional techniques in which a carbonaceous gas source is used to synthesize graphene. Several processes have been developed to synthesize graphene by this approach, such as electric arc, chemical conversion, CO reduction, CNT decompression and others [65]. However, of all processes reported in the literature, only chemical vapor deposition (CVD) and epitaxial growth appear to be attractive for the large-scale production of graphene. The top-down approach is based on an attack of bulk materials like graphite to break down its layers to form graphene sheets. Examples of the methods are micromechanical cleavage [69], exfoliation of graphite intercalated compounds (GIC) [70], solvent-base exfoliation, unzipping carbon nanotubes, and exfoliation or reduction of graphene oxide. This approach may be cost efficient although being limited to a lab scale and has limitations on quality control; also, it involves elevated investment and produces relatively low yields. Hence industries still search for economically favorable mass scale-up processes.

Graphite oxide can be synthesized by different methods, such as Brodie, Staudenmaier, or by Hummers method or improved Hummers method [71]. Major differences among all these methods are summarized in Table 4. The improved Hummers method has the lowest toxicity and several advantages with regard to the synthesized product [71].

**Figure 8:**
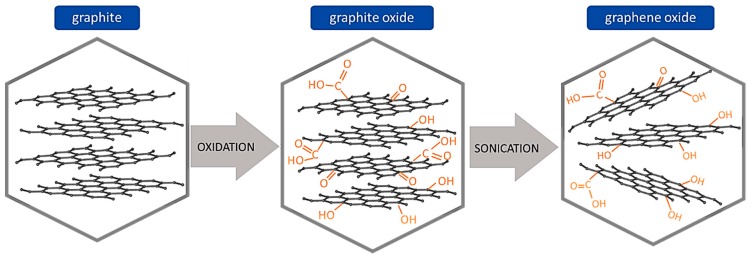
Representation of the general graphene oxide synthesis.

The general route to prepare GO involves two main steps (Figure 8): Firstly, graphite powder is oxidized to produce graphite oxide, and secondly, the bulk graphite oxide is exfoliated by sonication to form colloidal suspensions of monolayer, bilayer or few-layer GO sheets in different solvents [72]. Finally, solid GO can be recovered by drying the dispersion either in vacuum or atmospheric pressure at room temperature, freeze drying or at medium temperatures (50 and 65 °C) to prevent the thermal decomposition of GO. In the preparation of GO, the critical step is the selection of suitable oxidizing agents to oxidize graphite.

Several modifications based on the Hummers method have been proposed. For example, Kovtyukhova [73] added a pre-oxidized procedure using H_2_SO_4_, K_2_S_2_O_8_, and P_2_O_5_. The C/O ratio of the resultant oxidation product was 4.0/3.1, which was richer in oxygen than the graphite oxide prepared by the Hummers method. This method was defined as a typical modified Hummers method and has been cited by many researchers in recent years [74,75,76]. The improved Hummers method was proposed by Marcano [77]. By using KMnO_4_, H_2_SO_4_, and H_3_PO_4_ as the oxidizing agents, this method avoids the release of NO_x_ and produces a greater amount of hydrophilic oxidized graphite when compared to the original Hummers method [78].

In the case of graphene oxides, it is possible to obtain different surfaces material only use different methods of synthesis, since different methods lead to different ratios of C: O and different groups of oxygen [68,71,79,80,81].

A2—Functionalizations

REEs show a high affinity to O donors and although the graphene oxide displays good adsorption properties, due to its O-based surface functional group, in more complex aqueous environments it loses efficiency. In this context, a variety of methods for the graphene surface modification have been developed in order to give new properties to the material and to improve sorption efficiency. The functionalization plays a very important role, since it determines the material surface, and consequently the point zero charge (PZC) and the pH used in the batch experiments. Several ligands have been applied to REEs recovery with nanomaterials, such as ferrites NPs, and silica NPs. Ethylenediaminetetraacetic acid (EDTA) [82,83] diethylenetriaminepentaacetic acid (DTPA) [84], diglycolamic acid (DGA) [85,86,87], humic acids [88] and others chelating ligands [89] have been applied to carbon-based nanomaterials. These functionalizations can be achieved through the preparation of nanostructured silica-coated magnetite, followed by coating with the proposed functions. The resulting material tends to display higher sorption capacity of REEs and magnetic properties that simplify the separation process of the material in aqueous media.

A3—Recovery of Rare Earth Elements Using Graphene-Based Composites

Several articles describe the use of carbon nanocomposites for the recovery of REEs, either in batch or column experiments. Table 5 summarizes the recovery of REEs by GO nanocomposites, optimal experimental conditions being represented by shading. Almost all the studies were performed in Milli-Q water with the exception of Sun et al. [90] that used HClO_4_ (aq, 0.01 mol/L). This means that only conditions with no competitive ions were tested. Despite the numerous studies at different contact time between the nanomaterial and the rare earth solution, there are no published studies over 48 h. Most of the reported studies were performed at room temperature, although studies at higher temperature point to better rates of rare earth sorption.

A set of materials based on graphene oxide with different C:O ratios were collected. In addition to the GO, several functionalizations were performed to make the material more efficient, such as: Magnetite (Fe_3_O_4_) [91] with the purpose of affording magnetic properties to the material, making it more efficient for the REEs separation from solution, or polyaniline (PANI) [90] to increase the maximum adsorption capacity of the material. It is possible to find in the literature ratios mass of sorbent per volume of solution between the minimum of 40 mg/L and the maximum of 5000 mg/L, being 1000 mg/L the one most reported in the literature. The studies of Chen et al. [92] and W. B. Chen et al. [93] were the ones that reported the lowest value of sorbent mass/volume (40 mg/L), which was used in the recovery of Gd(III) and Y(III) ions, respectively, at pH 5.9 ± 0.1.

Most studies use mono-elemental systems and only Ashour et al. [94] and Su et al. [95] reported data from a multi-elemental system. Europium is the most studied element, although Cerium was reported by Fakhri et al. [96] and Farzin et al. [97], and Gadolinium, Scandium and Yttrium by W. Chen et al. [93], Kilian et al. [98] and W. B. Chen et al. [93], respectively. Ashour et al. [94] used a quaternary system with Lanthanum, Neodymium, Gadolinium and Yttrium; and Su et al. [95] studied a mixture of fifteen REEs. A wide variety of intervals of REE concentrations were used, between 10 µg/L [99] and 300,000 µg/L [98] were used, although the majority of the values were within 10,000–100,000 µg/L [90,91,92,93,94,96,100,101]. Lower concentrations, 10 and 50 µg/L of Eu(III) and Ce(III) ions were reported in Xie et al. [99] and Farzin et al. [97], respectively; moreover, Su et al. [95] used 10 µg/L for 15 elements in multi-elemental solution. Rare earth sorption is strongly pH dependent. In this way, several authors have tested pH between 2 and11 in order to search the optimal pH. The most used working pH is 6 [92,93,94,96,101]; the lowest pH was 2 [98,101] and the highest one was 7 [91,101,102]. A clear example that evidences this pH dependence on REEs sorption is the study of Li et al. [102] in which Eu(III) sorption by GO and Titanium phosphate modified GO composite (GTiP-2) varied from 7–10% at pH = 1, 20–50% at pH = 3.7 and 5, respectively, and 28–80% at pH = 7.3. At least two studies in the literature have reported achieving adsorption rates of approximately 100% using a pH of 5.5 [100] and 7 [101]. Finally, the material with the highest maximum adsorption capacity (qm) of REEs reported in the literature to date was PANI@GO with 250.74 mg/g achieved [90].

**Table 5:** Recovery of REEs using Graphene oxide (GO) composites and the respectively experimental conditions used as reported in the literature.

Ref., Year	Sorbent	Type of Water	Type of System	REEs (III)	[REEs]_0_ (µg/L)	pH	T (°C)	Time of Contact (h)	m(Sorbent)/ V(Solution) (mg/L)	q_m_ (mg/g) or REEs Adsorption (%)
[94], 2017	GO colloid	Ultrapure	Multi elements	La, Nd, Gd, Y	5 × 10^3^	6	r.t.	0.5	10 × 10^2^	La = 85.7 mg/g Nd = 189 mg/g Gd = 226 mg/g Y = 136 mg/g
[95], 2017	GO colloid	Ultrapure	Multi elements	La, Nd, Gd, Y	(5–50) 10^3^	3-8	5–45	0.02–2	10 × 10^2^	
[91], 2015	GO	Ultrapure	Mono element	Eu	10 × 10^3^ NaClO_4_ = 0.01 mol/L	4.5, 7	20	0–24	10 × 10^2^	90%, 89.7 mg/g
[92], 2015	MGO	Ultrapure	Mono element	Eu	10 × 10^3^ NaClO_4_ = 0.01 mol/L	4.5, 7	20	0–24	10 × 10^2^	80%, 70.2 mg/g
[92], 2015	GO e MGO	Ultrapure	Mono element	Eu	(1–50) ×10^3^	2–11	20, 40, 60	0–24	10 × 10^2^	
[101], 2012	GONS	Ultrapure	Mono element	Eu ^(1)^	51 × 10^3^ NaClO_4_ = 0.01 mol/L	2 4.5 6 7	25	48	2 × 10^2^	65%, 167.16 mg/g 161.29 mg/g 175.44 mg/g 100%
[101], 2012	GONS	Ultrapure	Mono element	Eu	51 × 10^3^ NaClO_4_ = 0.01 mol/L	2–11	25, 45, 65	48	2 × 10^2^	
[100], 2016	GO	Ultrapure	Mono element	Eu	10 × 10^3^ NaCl = 0.1, 0.01, 0.001 mol/L	5.5	20	0–24	5 × 10^2^	100%, 143 mg/g
[100], 2016	GO-OSO_3_H	Ultrapure	Mono element	Eu	10 × 10^3^ NaCl = 0.1, 0.01, 0.001 mol/L	5.5	20	0–24	5 × 10^2^	90%, 125 mg/g
[100], 2016	GO e GO-OSO_3_H	Ultrapure	Mono element	Eu	10 × 10^3^ NaCl = 0.1, 0.01, 0.001 mol/L	1–11	20	0–24	5 × 10^2^	
[92], 2014	GO colloid	Ultrapure	Mono element	Gd	12 × 10^3^	5.9 (2–11)	30	0.5	0.4 × 10^2^	287 mg/g
[93], 2014	GO colloid	Ultrapure	Mono element	Y	12 × 10^3^	5.9	30, 40	0.42	0.4 × 10^2^	190 mg/g
[99], 2016	GO	Ultrapure	Mono element	Eu	0.01 × 10^3^ NaCl = 0.01M	5.0, 2.7–7.3	r.t.	48	1 × 10^2^	78.0 mg/g, 97%
[99], 2016	GO	Ultrapure	Mono element	Eu	(0.01–100) × 10^3^	1–8 2,4,6	r.t.	48	1 × 10^2^	
[98], 2017	GO	Ultrapure	Mono element	Sc	300 × 10^3^	2 4	r.t.	4	50 × 10^2^	~ 95%, 36.5 mg/g 39.7 mg/g
[98], 2017	GO	Ultrapure	Mono element	Sc	(1–300) × 10^3^	1–5.5	r.t.	0.02–0.5	50 × 10^2^	
[96], 2017	30%Mo_4_W_8_@EDMG, 30%Mo_2_W_10_@EDMG	Ultrapure	Mono element	Ce	10 × 10^3^	6 (2–6)	20	0.08–3	17 × 10^2^	90.9 mg/g, 96.2 mg/g
[95], 2014	MPANI-GO	Ultrapure	Multi elements	Y, La, Ce, Pr, Nd, Sm, Eu, Gd, Tb, Dy, Ho, Er, Tm, Yb, Lu	0.01 × 10^3^	4	r.t.	0.33	4 × 10^2^	Y = 8.10, La = 15.5, Ce = 8.60, Pr = 11.1, Nd = 8.50, Sm = 7.70, Eu = 11.0, Gd = 16.3, Tb = 11.8, Dy = 16.0, Ho = 8.10, Er = 15.2, Tm = 10.4, Yb = 10.3, Lu = 14.9 mg/g
[96], 2014	MPANI-GO	Ultrapure	Multi elements	Y, La, Ce, Pr, Nd, Sm, Eu, Gd, Tb, Dy, Ho, Er, Tm, Yb, Lu	(0.00025, 0.0005, 0.001, 0.002, 0.01) × 10^3^	2–9	r.t.	0.02–0.25, 0.33	(0.25–20) × 10^2^	
[90], 2013	PANI@GO	HClO_4_ (aq) 0.01 mol/L	Mono element	Eu	15 × 10^3^	3	25	48	2.5 × 10^2^	251 mg/g
[97], 2017	TGA/CdTeQDs/Fe3O4/rGONS	Distilled	Mono element	Ce	0.05 × 10^3^ (1–100) × 10^3^	5.0	35	0.17	7 × 10^2^	95% 56.8 mg/g
[102], 2017	TGA/CdTeQDs/Fe3O4/rGONS	Distilled	Mono element	Ce	(1–100) × 10^3^	2-8	35	0.02–0.25	(2–9) × 10^2^	
[102], 2014	GTiP-1	Ultrapure	Mono element	Eu	100 × 10^3^	1 3.7 5.5 7.3	25	2	10 × 10^2^	~ 3.0% ~ 32% 35% ~ 72%
[103], 2014	GTiP-2	Ultrapure	Mono element	Eu	100 × 10^3^	1 3.7 5.5 7.3	25	2	10 × 10^2^	~ 10% ~ 45% 50% ~ 80%
[103], 2014	GO	Ultrapure	Mono element	Eu	100 × 10^3^	1 3.7 5.5 7.3	25	2	10 × 10^2^	~ 7.0% ~ 20% 20% ~ 28%
[103], 2014	GO, GTiP-1, GTiP-2	Ultrapure	Mono element	Eu	(5–200) × 10^3^ Na^+^ = 1, 10, 100, 1000 mM	1.7, 3.7, 5.5, 7.3	25	2, 4	10,000 × 10^2^	

^(1)^ Adsorptions experiments were conducted under N_2_ conditions. r.t. means room temperature. The ultrapure water was provided by Milli-Q system. Note that the optimal experimental conditions are represented by shading and the other conditions tested and described in the papers are represented on a white background (without shading).

##### B. Carbon Nanotubes

Carbon nanotubes (CNTs) are unique nano-structures with remarkable electronic and mechanical properties, either due to their close relationship with graphene, or because of their one-dimensional appearance. From the structural point of view, carbon nanotubes are divided into two main types of carbon nanotubes: Single-walled carbon nanotubes (SWCNTs), which can be considered as a single sheet of graphene rolled on itself to form a cylindrical tube, and multi-walled carbon nanotubes (MWCNTs), which consist of a set of concentric nanotubes stabilized by van der Waals forces [66]. The presence of concentric graphene sheets on MWCNTs enhance the interaction with the analytes [38].

**Figure 9:**
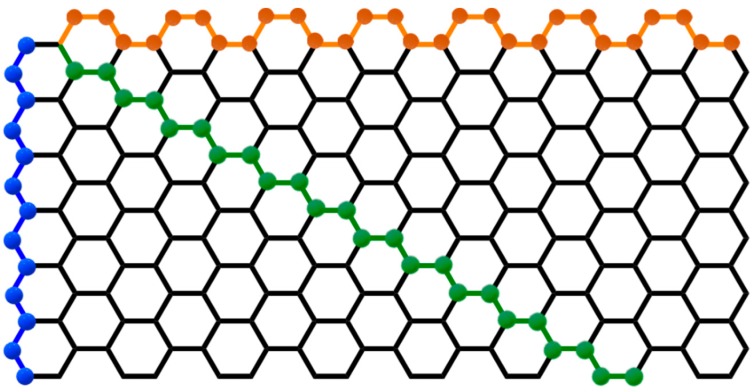
Classification of single-walled carbon nanotubes with distinct geometry and properties: Armchair (**orange**), zigzag (**blue**) and chiral (**green**).

The structure of the single-walled carbon nanotubes is determined by how close they are to themselves in the hexagonal network of graphene. These nanotubes can have three distinct forms (Figure 9), designated as an armchair, zigzag and chiral. The three arrangements present different electrical conduction properties, which result in the exceptional electronic properties of single wall carbon nanotubes. All carbon nanotubes of the armchair type are conductors, whereas the zigzag and chiral type can be conductors or semiconductors [66].

**Figure 10:**
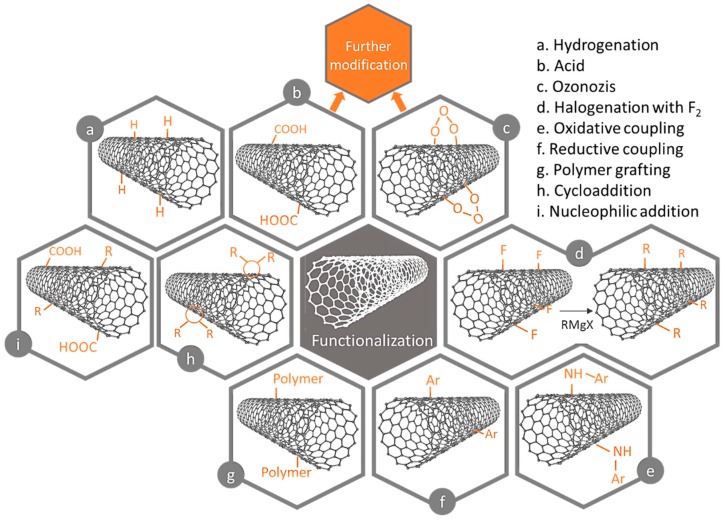
Overview of surface functionalization methods of over carbon nanotubes (CNTs).

Another important property is the carbon nanotubes insolubility in most liquids, such as water, polymer resins and in almost all solvents. So, to facilitate and standardize the dispersion of nanotubes in liquids, functional groups or polar molecules can be incorporated into the walls (Figure 10) without significantly altering their properties [66].

B1—Production of CNTs and Its Functionalizations

**Figure 11:**
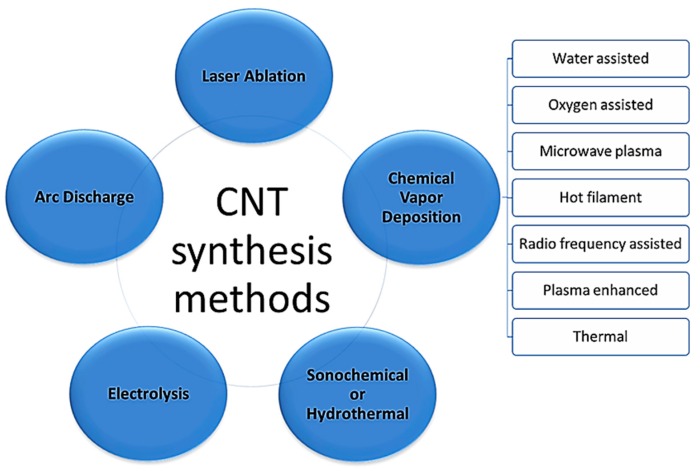
Different approaches to CNTs synthesis.

Since its discovery, the methods of synthesis of carbon nanotubes have been continuously optimized, in order to obtain pure nanotubes in enough quantities. The main techniques of synthesis (Figure 11) can be divided into [66]: (i) High temperature methods, which include electric arc discharge and laser ablation; and (ii) methods at moderate temperatures, including chemical vapor deposition assisted by a catalyst [103]. This has been widely diffused and optimized, allowing even large-scale production [104]. Among these techniques, the most widely used for producing nanotubes is the electric arc discharge, is also used for the preparation of fullerene molecules. Either in single-walled tubes or multiple-walled tubes, process parameters, such as flow, gas pressure, and metal concentration need to vary to obtain the highest yield of carbon nanotubes [66].

**Figure 12:**
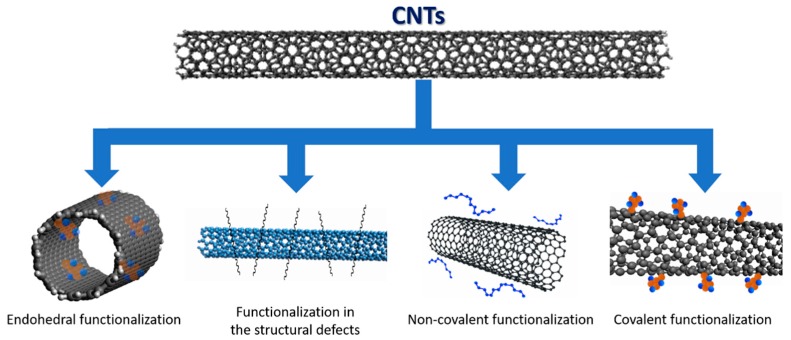
Types of functionalization methods of carbon nanotubes.

Functionalization may be non-covalent (physical functionalization) or covalent (chemical functionalization), as represented in Figure 12. The non-covalent functionalization of nanotubes is based on the use of surfactants capable of making this material “soluble” in water; this process results from weak van der Waals interactions and π-π type interactions. Covalent functionalization is based on the establishment of covalent bonds of functional ligands to the structures of the carbon nanotubes, which can occur at the ends or the tube walls. Functionalization in structural defects occurs through chemical transformations. Finally, the endohedral functionalization is the filling of the nanotubes with atoms or molecules of small dimensions. This type of modification, either by covalent or non-covalent functionalization, changes the surface properties, directly influencing the sorption capacity of the carbon nanotubes.

B2—Recovery of Rare Earth Elements by Carbon Nanotubes

Table 6 shows the studies of the recovery of REEs using carbon nanotubes composites. All the studies were performed in Milli-Q or distilled water, with the exception of only one study performed by Yadav et al. [105] that used HCl (aq, 0.5 M). Regarding the contact time between the nanocomposite and the rare earth solution, there was a wide range of times used, although no studies published exceeded 96 hours and the majority had 2–4 h duration [98,105,106,107,108]. Temperature was tested between 20 and 65 °C, although most of the reported studies were performed at 30 °C [105,106,107].

In most studies, the oxidized multi-walled carbon nanotubes (MWCNTs-oxidized) were chosen, since they are a more efficient and cheaper material when compared to the single-walled carbon nanotubes (SWCNTs-oxidized). In addition, some studies showed sorption experiments using CNTs with several functionalizations to improve even more their efficiency or to introduce other properties, such as magnetite (Fe_3_O_4_) [109] with the purpose of affording magnetic properties to the material, or chitosan [108] to increase the maximum adsorption capacity of the material. It is possible to find, in the literature, ratios mass of sorbent per volume of solution from 600 to 100,000 mg/L for the REEs recovery, however, the most reported values were 600, 1000 and 5000 mg/L. The studies of Fan et al. [110], Chen et al. [109] and Chen et al. [111] were the ones that reported the lowest value of sorbent mass/volume (600 mg/L), which was used in the recovery of Eu(III) in mono-elemental solutions and at a pH between 5 and 6. Finally, the material with the highest maximum adsorption capacity (qm) of REEs reported in the literature to the date was mIIP-CS/CNT composite with 121.51 mg/g achieved [108].

More studies used multi-elemental systems with CNTs composites than GO composites. The REEs studies in mono-elemental systems were scandium and europium, and in multi-elemental systems cerium, samarium, lanthanum, dysprosium, terbium, lutetium and gadolinium ions; yttrium was studied in both types of system. The two elements most studied were lanthanum and europium. Sorption studies in multi-elemental systems were limited to a maximum of three elements by Tong et al. [112] and Yadav et al. [105]. A wide variety of concentration intervals is published (Table 6), from 30 µg/L [111] to 1,000,000 µg/L [105], being the most used concentrations between 10,000 and 40,000 µg/L. The lower concentrations, namely 30 and 61 µg/L of Eu(III), in mono-elemental solutions, were reported by Chen et al. [111] and Chen et al. [109], respectively; moreover, K. Li et al. [108], Koochaki-Mohammadpour [107] and Behdani et al. [106] used 10 000 µg/L for different REEs like La(III) and Dy(III) or Ce(III) and Sm(III), in multi-elemental solutions. Maximum adsorption values of REEs by CNTs composites are highly dependent on the chosen working pH, affecting the surface charge and, consequently, the sorption of metal ions on CNTs. In general, increasing pH leads to the increase of metal ions sorption; this occurs because, at pH superior to pH_PZC_ (point of zero charge), the positively-charged metal ions can be adsorbed on the negatively-charged oxidized CNTs [38]. In this way, the pH interval 5–7 were tested to find out the optimal pH and/or the working pH. The most used working pH is 5 [106,107,109,110,112]; also, the lowest pH used was 1.5 [98,112] whereas the highest working pH chosen was 8 [106,110]. Nevertheless, at least two studies in the literature have achieved adsorption rates of approximately 100% using a pH of 5 [106] and 5.5 [109].

**Table 6:** Recovery of REEs using CNTs and the respectively experimental conditions used as reported in the literature.

Ref., Year	Sorbent	Type of Water	Type of System	REEs (III)	[REEs]_0_ (µg/L)	pH	T (°C)	Time of Contact (h)	m (sorbent)/ V(solution) (mg/L)	q_m_ (mg/g) or REEs Adsorption (%)
[98], 2017	CNTs-COOH	Ultrapure	Mono element	Sc	300 × 10^3^	2 4	r.t.	4	50 × 10^2^	37.9 mg/g 42.5 mg/g
[98], 2017	CNTs-COOH	Ultrapure	Mono element	Sc	(1–300) × 10^3^	1–5.5	r.t.	0.02–0.5	50 × 10^2^	-
[106], 2013	MWCNTs-oxidized	Distilled	Multi elements	Ce	20 × 10^3^ 20 × 10^3^ 10 × 10^3^	5	30	2	12 × 10^2^ 10 × 10^2^ 10 × 10^2^	~ 87% ~ 82% ~ 97%
[107], 2013	MWCNTs-oxidized	Distilled	Multi elements	Sm	20 × 10^3^ 20 × 10^3^ 10 × 10^3^	5	30	2	12 × 10^2^ 10 × 10^2^ 10 × 10^2^	~ 98% ~ 95% ~ 100%
[107], 2013	MWCNTs-oxidized	Distilled	Multi elements	Ce, Sm	(10, 20, 50, 75, 100, 150, 200) × 10^3^	2–8	30, 40, 50, 60	0.08, 0.17, 0.25, 0.33, 0.5, 0.67, 0.83, 1, 1.25, 1.5, 2	(2, 4, 6, 8, 10, 12) × 10^2^	-
[107], 2014	MWCNTs-oxidized	Distilled	Multi elements	La	20 × 10^3^ 20 × 10^3^ 10 × 10^3^	5	30	2	12 × 10^2^ 10 × 10^2^ 10 × 10^2^	80% 80% 93%
[108], 2014	MWCNTs-oxidized	Distilled	Multi elements	Dy	20 × 10^3^ 20 × 10^3^ 10 × 10^3^	5	30	2	12 × 10^2^ 10 × 10^2^ 10 × 10^2^	98% 97% 98%
[108], 2014	MWCNTs-oxidized	Distilled	Multi elements	La, Dy	(10–200) × 10^3^	2–6	30, 40, 50, 60	0.08, 0.17, 0.25, 0.33, 0.5, 0.67, 0.83, 1, 1.25, 1.5, 2	(2–12) ×10^2^	
[112], 2011	TA-MWCNTs	Distilled	Multi elements	La Tb Lu	40 × 10^3^	5	20	1	50 × 10^2^	5.35 mg/g, 8.55 mg/g, 3.97 mg/g
[113], 2011	TA-MWCNTs	Distilled	Mono element	La	40 × 10^3^	5	20	1	50 × 10^2^ (with 0.12 × 10^2^ being TA)	75%
[113], 2011	TA-MWCNTs	Distilled	Multi elements	(La, Tb, Lu)	40 × 10^3^	1.5–4	20	1	50 × 10^2^	0.4–6.0 mg/g
[113], 2011	TA-MWCNTs	Distilled	Multi elements	(La, Tb, Lu)	(5–50) × 10^3^	1.5–7	20	0.08–2	(20–200) × 10^2^	-
[110], 2009	MWCNTs-oxidized	Milli-Q	Mono element	Eu	0.99 × 10^3^	5 (2–8)	25	96	6 × 10^2^	90.0%
[109], 2009	MWCNTs/Fe_3_O_4_ composite	Milli-Q	Mono element	Eu ^a^	0.061 × 10^3^ NaClO_4_ = 0.1 mol/L	5.5	25	48	6 × 10^2^	~ 100%
[110], 2009	MWCNTs/Fe_3_O_4_ composite	Milli-Q	Mono element	Eu ^a^	0.61 × 10^3^, 6.1 × 10^3^	2.5–7	25	48	6 × 10^2^	-
[105], 2015	PES/PVA/MWCNT/ D2EHPA beads	HCl (aq, 0.5 mol/L)	Mono element	Y	1000 × 10^3^	–	30	8	1000 × 10^2^	95%
[106], 2015				Y	(80–3300) × 10^3^	–	30–65	0–8	1000 × 10^2^	44.1 mg/g
[106], 2015			Multi elements	Y Sm La	100 × 10^3^	–	30	4	1000 × 10^2^	94% 82% 30%
[106], 2015	PES/PVA/MWCNT/ D2EHPA beads	HCl (aq, 0.5 mol/L)	Multi elements	Y, Sm, La	(150–1000) × 10^3^	–	30	0–8	1000 × 10^2^	-
[111], 2008	MWCNTs-oxidized	Distilled	Mono element	Eu	0.03 × 10^3^ NaClO_4_ = 0.001, 0.01, 0.1 mol/L	6 (2–7)	25	48	6 × 10^2^	98% for all the ionic strengths
[108], 2015	mIIP-CS/CNT composite	Distilled	Multi elements	Gd ^b^	10 × 10^3^	7	20 33 43	4	20 × 10^2 c^	79.5 mg/g 109 mg/g 122 mg/g
[109], 2015	mNIP-CS/CNT composite	Distilled	Multi elements	Gd ^b^	10 × 10^3^	7	33	4	20 × 10^2 c^	96.2 mg/g
[109], 2015	mIIP-CS/CNT and mNIP-CS/CNT composites	Distilled	Multi elements	Gd ^b^	(2, 10, 50, 100, 200) × 10^3^	2–7	20, 33, 43	0.05–8	20 × 10^2 c^	-

^a^ Adsorptions experiments under N_2_ conditions. ^b^ Gd^3+^ adsorption experiments with two competitive ions (La^3+^ and/or Ce^3+^). ^c^ 10 mg of IIP-CS/CNT (or NIP-CS/CNT) and 30 mg of SiO_2_@Fe_3_O_4_ were added into a vial, which contained 20 mL of REEs. Optimal experimental conditions are represented by shading and the other conditions tested and described in the papers are represented on a white background (without shading).

##### C. Other Carbon Materials

**Activated carbon** is a material composed mostly of carbon, very porous, and it is considered one of the adsorbents with a greater capacity of adsorption. Its main characteristic is its high internal surface area developed during activation, formed by thousands of pores classified in micro, meso and macropores. Activated carbons are typically used to purify or separate gas and liquid mixtures [113,114] because of their high adsorption capacity; however, commercially activated carbons generally have high costs which may limit their use. The most common forms in which they are marketed are powdered activated carbon (PAC) and granular activated carbon (GAC). GAC is most frequently used in the removal of water pollutants, since it allows a continuous process in columns of fixed and immobile beds through which the fluid passes and is purified [114].

A **fullerene** is a molecule of carbon in the form of a hollow sphere, ellipsoid, tube, and many other shapes. Basically, fullerenes are closed hollow cages made of sp^2^-hybridized carbon atoms arranged into 12 pentagons and a calculable number of hexagons that depends on the total number of carbon atoms. C60 is the most abundant and the most widely studied, to date. C60 and other larger fullerenes (C70, C76, C82, and C84) can be viewed as a carbon nanoallotrope with hybridization between sp^2^ and sp^3^. The presence of pentagons is essential, introducing curvature and, hence, allowing closing of the cage [113].

**Carbon dots** or C-dots are quasi-spherical carbon nanoparticles with diameters of 2−10 nm that have high oxygen contents and consist of combinations of graphitic and turbostratic carbon in various volumetric ratios. The most characteristic and significant property of C-dots is relatively strong photo-luminescence, which depends on their size, the excitation wavelength, and the surface functionalization [113].

**Carbon nanofibers (CNFs)** are described as a non-continuous 1D carbon nanoallotrope of cylindrical or conical shape, consisting of stacked and curved graphene sheets arranged in various ways. They are frequently described as sp^2^-based linear filaments with a diameter ranging from 50 to 200 nm and a high aspect ratio exceeding 100. CNFs have special surface morphology, steady structure characteristics, surface properties which can be modified through chemical treatments to achieve a specific goal and they are also easily available on a large scale. For these reasons, CNFs may have great analytical potential as an effective SPE adsorbent [113].

**Carbon black (CB)** is a material produced by the incomplete combustion of heavy petroleum products (such as FCC tar and ethylene cracking tar) with the addition of a small amount of vegetable oil. Carbon black is a form of paracrystalline carbon that has a high surface-area-to-volume ratio, although lower than activated carbon. His mainly used as a reinforcing filler in tires and other rubber products; as color pigments in plastics, paints, and inks, and as SPE adsorbent for chemical product recovery [115].

C1—Recovery of REEs Using Other Carbon Materials

Table 7 represented the studies of the REEs recovery using the other types of carbon materials which do not belong to the graphene or carbon nanotubes families (activated carbon, fullerenes, carbon dots, mesoporous carbon, carbon nanofibers and carbon black). Again, this review is only focused on batch experimental studies whereby there are a few studies in the literature that are not been explored in this review because they are column experiments [38,116,117,118]. However, it was possible to verify that carbon nanotubes and carbon nanofibers are the most used materials for column experimental studies.

All the studies were performed in Milli-Q, with the exception of two studies by Gad and Awwad [119] that used laboratory wastewaters in addition to Milli-Q water and Marwani et al. [120] that used distilled water, tap water, lake water and seawater. Regarding the contact time between the nanocomposite and the rare earth solution, there was a wide range of times, nevertheless, there are no studies published over 48 h [101] and the majority of the studies performed had a duration time of 1 and 24 h [115,119,120]. A wide range of temperature (20–80 °C) was, although most of the reported studies were performed at room temperature, 25 °C.

Activated carbon is the most used material, with various types of functionalization. Ratios mass of sorbent per volume of solution were between 3 mg/L and 5000 mg/L and the most reported ratio was 1000 mg/L. The study of Smith et al. [115] reported the lowest values of sorbent mass/volume (3 and 25 mg/L), which was used in the recovery of La(III), Ce(III), Nd(III), Sm(III) and Y(III) in multi-elemental solutions and at neutral pH. Finally, the material with the highest maximum adsorption capacity (qm) of REEs reported in the literature was oxygen and phosphorus functionalized nanoporous carbon with 335.5 mg/g and 344.6 mg/g achieved of Nd and Dy, respectively, at pH 6.1 and 6.6 in multi-elemental solution [121]. However, the best removal rate achieved was 99.6% of La by BETADHBA functionalized activated carbon at pH 6 [121].

It was found more studies using multi-elemental systems with these materials than multi-elemental systems with GO composites. La(III), Nd(III) and Eu(III) were the elements most studied. Most of the studies have used concentrations from 0.3 µg/L [122] to 300,000 µg/L [98], although most of the values used were from 50,000 to 100,000 µg/L. The studies of Perreault et al. [122] used the lower concentrations values, namely 70 and 0.3 µg/L of Sm(III) and La(III), respectively. Sorption is highly dependent on the chosen working pH. The most used working pH were 5 and 6; the lowest pH used was 2 [98,119,122] and the highest working pH was 7 [115,121]. A clear example that evidences this pH dependence on REEs adsorption is the study of Gad and Awwad [119] which got an increase of adsorption capacity from 20 mg/g at pH 2 for 32 mg/g at pH 5, 47 mg/g at pH 6 and 50 mg/g of Eu at pH 7. Furthermore, the study of Marwani et al. [120] demonstrates an increase of sorption rate from 40% of La(III) at pH 4 to 85% at pH 5 and 99.60% at pH 6. This study reported the best sorption rate in the literature. Finally, the material with the highest maximum adsorption capacity (qm) of REEs reported in the literature to date was oxide and phosphorous functionalized nanoporous carbon with 344.6 mg/g of Dy achieved [121]; and, the best maximum adsorption capacity by an activated carbon was BETADHBA functionalized activated carbon (AC-BETADHBA) with 144.80 mg/g for La(III) [120].

## 5. Conclusions Remarks and Perspectives

Technology has never been so much dependent on electronic devices as it is today, which means a strong REEs dependency. Electronic devices become obsolete too quickly generating great amounts of e-waste annually, creating the need for a strategy to deal with this type of waste material. the incorrect treatment and storage of e-waste can cause serious damage to the environment with a result of REEs or even more toxic metals (such as Hg and Pb) in aquatic environments. It is hence necessary to create and promote the recycling of e-waste.

There are several techniques to the recovery of rare earth elements, however, the most developed techniques are very harmful to the environment. New, greener and more efficient methods are needed. Carbon-based nanocomposites have been increasingly used for the recovery of metals, namely rare earth, due to their satisfactory physical and chemical properties, such as large surface area and a large number of oxygen groups to sorption. In addition, it is possible to incorporate functionalization in their defects that allow to improve the sorption efficiency or to provide them additional properties, such as magnetite, which make easy to remove the material from the solution by application of an external magnetic field. Carbon-based materials reported in the literature as being good sorbents were groups in this review in three categories: Graphene-based materials, carbon nanotubes, and other carbon materials that include activated carbon, fullerene, C-Dots, carbon black, mesoporous carbon, and carbon nanofibers. REE removal efficiency reported for those materials is highly variable, depending on key factors, such as pH of the solution, the mass of the sorbent, and time of contact between the contaminated solution and the sorbent. Only a few studies have tested the effect of competitive ions in solution on the REE sorption efficiency. The influence of ionic strength was almost neglected in the works published so far. Furthermore, almost all the experiments were carried out in Milli-Q water, with non-realistic concentrations and high doses of sorbent.

This review intended to summarize the progress on the thematic of REE removal by carbon-based materials, and to identify possible gaps. To prove the advantages of using carbon-based nanomaterials for REEs recovery from real industrial wastewaters and/or from end-of-life products a long and prosper way needs to be done. Tests with multi-element contaminated solutions, complexed situations with solutions of different ionic strengths and pH, and approaches to realistic conditions are recommended.

## Figures and Tables

**Table 1:** Overview of REEs applications and end uses.

Element (Symbol) *	Application and End Use	Ref.
Sc	aerospace framework/components, high-intensity street lamps/additive in metal-halide lamps and mercury vapor lamps, radioactive tracing agent in oil refineries.	[15,19,22,27,28]
Y	TV sets, cancer treatment drugs, enhances strength of alloys, lasers, high temperature superconductors, microwave filters, energy-efficient light bulbs, spark plugs, gas mantles	[15,19,22,27,29]
La	camera lenses, battery-electrodes, hydrogen storage, fluid catalysts for oil refineries	[15,20,22,27,28]
Ce	catalytic converters, colored glass, steel production, chemical oxidizing agent	[15,19,22,27,28]
Pr	magnets, welding goggles, lasers	[15,22,27,30,31,32]
Nd	permanent magnets, microphones, electric motors of hybrid automobiles, lasers	[15,19,22,27,30,31,32,33,34]
Pm	nuclear batteries	[15,22]
Sm	cancer treatment, nuclear reactor control rods, X-ray lasers, masers, magnets	[15,19,22,27]
Eu	color TV screens, fluorescent glass, genetic screening tests	[15,19,22,27,29,35,36,37]
Gd	shielding in nuclear reactors, nuclear marine propulsion, increases durability of alloys	[15,19,22,27,28]
Tb	TV sets, fuel cells, sonar systems, florescence lamps, lasers	[15,16,19,22,27,36]
Dy	commercial lighting, hard disk devices, transducers, magnets	[15,19,22,27,30,31,32,33,34]
Ho	lasers, glass coloring, high-strength magnets	[15,16,19,22]
Er	glass colorant, signal amplification for fiber optic cables, metallurgical uses	[15,16,19,22]
Tm	high efficiency lasers, portable X-ray machines, high temperature superconductor	[15,16,19,22]
Yb	improves stainless steel, lasers, ground monitoring devices	[15,16,19,22]
Lu	refining petroleum, LED light bulbs, integrated circuit manufacturing	[15,16,19,22]

* REEs are listed in order of increasing atomic number.

**Table 2:** Overview of the distribution of REEs usage (in % of rare earth oxides) in different applications, in 2012. Data source: EU report on Critical Raw Materials [20].

REEs	Magnets	Batteries	Metallurgy	Catalysts	Polishing	Glass	Phosphors	Ceramics	Others	Total
La	0	26	10	45	1	5	1	1	9	100
Ce	0	3	19	18	36	12	4	1	8	100
Pr	73	0	4	0	2	0	12	7	2	100
Nd	89	0	2	2	0	1	1	4	0	100
Sm	97	0	0	0	0	0	0	0	3	100
Eu	0	0	0	0	0	0	96	0	4	100
Gd	35	0	28	0	0	0	23	0	14	100
Tb	24	0	0	0	0	0	71	0	5	100
Dy	98	0	0	0	0	0	0	0	2	100
Er	0	0	0	0	0	72	25	0	3	100
Y	0	0	0	0	0	0	79	21	0	100
Ho, Tm, Yb, Lu	0	0	0	0	0	0	0	0	100	100
All REEs	20	8	11	20	15	7	9	3	6	100

**Table 3:** The use of hydrometallurgical methods for the recovery of REEs from end-of-life products, according to the studies published in 2016 [24].

REE	Recycle	Method used	% Recovery	Reference
REE	Permanent Magnet (Review)	Hydro and Pyrometallurgy	NA	[43]
La, Ce	Oil refining Catalyst (Review)	Hydrometallurgy	NA	[48]
La, Nd	NiMH magnets (Review)	Hydrometallurgy	NA	[49]
Ce, Pr, Nd, Sm	NiMH magnets	Hydrometallurgy	98.1 (Nd), 95.5 (Sm), 95.5 (Pr), 89.4 (Ce)	[50]
Pr	Fuel cell catalyst	Hydrometallurgy	76	[51]
Pr, Nd	Permanent Magnet	Vacuum Induction melting, hydrolysis and magnetic separation	93 (99.7% purity)	[52]
Pr, Nd, Dy	Motors	Hydrometallurgy	82 (99% purity)	[53]
Eu, Y	Phosphor (lamps)	Hydrometallurgy	100 (99.9% purity)	[54]
Eu, Y	Fluorescent lamp	Hydrometallurgy	99.9	[55]
Eu, Tb, Y	Phosphor (lamps)	Mechanical Activation and leaching	89.4 (Tb), 93.1 (Eu), 94.6 (Eu)	[56]

**Table 4:** List of the advantages and disadvantages of different synthesis methods for graphite oxide. Data source: [71].

Method	Oxidants	Toxicity	Advantages	Disadvantages
Brodie Method	KClO_3_, HNO_3_	Yes	-	Weak acidity. Soft dispersibility in basic solutions. Small size, limiting thickness and providing an imperfect structure.
Staudenmaier Method	KClO_3_ (NaClO_3_), HNO_3_, H_2_SO_4_	Yes	-	Time-consuming and dangerous method. Addition of KClO_3_ generally takes longer than a week and CO_2_ is evolved, thus making necessary to remove an inert gas. The risk of explosions is a constant danger.
Hummers Method	KMnO_4_, H_2_SO_4_, NaNO_3_	No (NOx is released)	Higher oxidation degree than that obtained in Brodie or Staudenmaier Methods.	It is still considered than the oxidation is incomplete. Separation and purification processes are tedious process. Highly time-consuming process.
Modified Hummers Method	KMnO_4_, H_2_SO_4_, NaNO_3_, KMnO_4_, H_2_SO_4_	No (NOx is released)	Improved level of oxidation and, therefore, product performance.	Separation and purification processes are tedious process. Highly time-consuming process.
Improved Hummers Method	KMnO_4_, H_2_SO_4_, H_3_PO_4_	No	Defects in the basal plane are reduced. Larger amount of oxidized graphite is provided. The degree of reduction provides an equivalent level of conductivity when compared to other methods. Best process yield compared to Brodie, Staudenmaier and Hummers method. Environmentally friendly, toxic gases are not generated during the preparation. The product has a more organized structure compared to graphite oxide obtained by Brodie and Staudenmaier methods.	Separation and purification processes are tedious process. Highly time-consuming process.

**Table 7:** Recovery of REEs using other carbon materials (Activated Carbon, Fullerene, C-Dots, Carbon Black, Mesoporous Carbon, Carbon nanofibers) and the respectively experimental conditions used as reported in the literature.

Ref., Year	Sorbent	Type of Water	Type of System	REEs (III)	[REEs]_0_ (µg/L)	pH	T (°C)	Time of Contact (h)	m(sorbent)/ V(solution) (mg/L)	qm (mg/g) or REEs Adsorption (%)
[101], 2012	AC (Activated Carbon)	Ultrapure	Mono elemental	Eu	10 × 10^3^ NaClO_4_ = 0.01 mol/L	4.5	25	48	2 × 10^2^	20.0 mg/g
[98], 2017	AC-COOH	Ultrapure	Mono elemental	Sc	300 × 10^3^	2	r.t.	4	50 × 10^2^	2.10 mg/g
	AC-COOH	Ultrapure	Mono elemental	Sc	300 × 10^3^	4	r.t.	4	50 × 10^2^	2.20 mg/g
	AC-COOH	Ultrapure	Mono elemental	Sc	(1–300) × 10^3^	1–5.5	r.t.	0.02–4	50 × 10^2^	
[115], 2016	F-CCB (Functionalized commercial carbon black)	Ultrapure	Multi elemental	La, Ce, Nd, Sm, Y	100 × 10^3^	neutral pH	25	24	0.25 × 10^2^	La = 15%, Ce = 41%, Nd = 23%, Sm = 14%, Y = 17%
	F-CCB (Functionalized commercial carbon black)	Ultrapure	Multi elemental						(0.03, 0.05, 0.15) × 10^2^	La = 12%, 13%, 14% Ce = 36%, 36%, 35% Nd = 10%, 12%, 16% Sm = 10%, 10%, 13% Y = 12%, 13%, 13%
	RTCB (Recycled tire carbon black)	Ultrapure	Multi elemental						0.25 × 10^2^	La = 28%, Ce = 68%, Nd = 34%, Sm = 41%, Y = 28%
	RTCB (Recycled tire carbon black)	Ultrapure	Multi elemental	La, Ce Nd, Sm, Y	100 × 10^3^	neutral pH	25	24	(0.03, 0.05, 0.15) × 10^2^	La = 3.5%, 6.0%, 18% Ce = 11%, 15%, 42% Nd = 5.0%, 7.5%, 22% Sm = 5.5%, 9.0%, 26% Y = 3.5%, 6.0%, 18%
	F-AC (Functionalized activated carbon)	Ultrapure	Multi elemental	La, Ce Nd, Sm Y	100 × 10^3^	neutral pH	25	24	0.25 × 10^2^ (0.03, 0.05, 0.15) × 10^2^	La = 7.5%, Ce = 12%, Nd = 31%, Sm = 7.5%, Y = 12.5% La = 1.5%, 2.5%, 6.5% Ce = 2.5%, 8.0%, 11% Nd = 9.0%, 17%, 24% Sm = 0%, 7.5%, 5% Y = 6.0%, 9.0%, 11%
	CCB (commercial carbon black)	Ultrapure	Multi elemental	La, Ce Nd, Sm Y	100 × 10^3^	neutral pH	25	24	(0.15, 0.25) × 10^2^	La = 2.5%, 2.5% Ce = 1.0%, 1.0% Nd = 5.0%, 8.0% Sm = 1.0%, 2.5% Y = 2.5%, 3.0%
	CCB (commercial carbon black)								(0.03, 0.05) × 10^2^	La= 2.5%, Ce = 1.0%, Nd = 5.0%, Sm = 1.0%, Y = 2.5%
	AC								(0.15, 0.25) × 10^2^	La =1.0%, Ce =1.0%, Nd = 12.5%, Sm = 0%, Y = 0%
[116], 2016	AC	Milli-Q	Multi elemental	La Ce Nd Sm Y	100 × 10^3^	neutral pH	25	24	(0.03, 0.05) × 10^2^	La = 1.0%, 1.5% Ce = 1.0%, 1.0% Nd = 7.5%, 8.0% Sm = 0%, 1.0% Y = 1.5%, 1.0%
	RTCB (Recycled tire carbon black)	Ultrapure	Multi elemental	La Ce Nd Sm Y	20 × 10^3^	neutral pH	80	1	0.5 × 10^2^	La = 40%, Ce = 95%, Nd = 75%, Sm = 80%, Y = 63%
								2	0.5 × 10^2^	La = 45%, Ce = 95%, Nd = 80%, Sm = 82%, Y = 72%
								12	0.5 × 10^2^	La = 75%, Ce = 95%, Nd = 91%, Sm = 95%, Y = 90%
							25	1	0.5 × 10^2^	La = 25%, Ce = 85%, Nd = 68%, Sm = 60%, Y = 48%
								2	0.5 × 10^2^	La = 45%, Ce = 90%, Nd = 70%, Sm = 73%, Y = 60%
[116], 2016	RTCB (Recycled tire carbon black)	Ultrapure	Multi elemental	La, Ce Nd, Sm Y	20 × 10^3^	neutral pH	25	12	0.5 × 10^2^	La = 60%, Ce = 95% Nd = 83%, Sm = 88%, Y = 77%
					100 × 10^3^		40	24	0.05 × 10^2^	La = 5.5%, Ce = 23%, Nd = 9.0%, Sm = 9.0%, Y = 9.0%
					100 × 10^3^		60	24	0.05 × 10^2^	La = 7.5%, Ce = 25% Nd = 16%, Sm = 16%, Y = 16%
					100 × 10^3^		80	24	0.05 × 10^2^	La = 13%, Ce = 30% Nd = 20%, Sm = 20%, Y = 21%
					100 × 10^3^		40	24	0.25 × 10^2^	La = 29%, Ce = 75% Nd = 40%, Sm = 40%, Y = 40%
					100 × 10^3^		60	24	0.25 × 10^2^	La = 323%, Ce = 81%, Nd = 50%, Sm = 55%, Y = 50%,
					100 × 10^3^		80	24	0.25 × 10^2^	La= 48%, Ce = 84% Nd = 58%, Sm = 60%, Y = 60%
					100 × 10^3^		40	24	0.5 × 10^2^	La= 45%, Ce = 85% Nd = 65%, Sm = 68%, Y = 60%
					100 × 10^3^		40	24	0.5 × 10^2^	La= 45%, Ce = 85% Nd = 65%, Sm = 68%, Y = 60%
					100 × 10^3^		40	24	0.5 × 10^2^	La = 45%, Ce = 85% Nd = 65%, Sm = 68%, Y = 60%
[116] 2016	RTCB (Recycled tire carbon black)	Ultrapure	Multi elemental	La, Ce, Nd, Sm, Y	100 × 10^3^	neutral pH	60	24	0.5 × 10^2^	La = 52%, Ce = 90% Nd = 70%, Sm = 72%, Y = 70%
							80			La = 69%, Ce = 90% Nd = 75%, Sm = 75%, Y = 75%
	F-CCB, RTCB, F-AC, AC	Ultrapure (Shaker: 200 rpm)	Multi elemental	La, Ce, Nd, Sm, Y	(100–200) × 10^3^	neutral pH	25, 40, 60, 80	1–24	(0.25–0.5) × 10^2^	
[119], 2007	H-APC AC (HPO_4_-APC activated carbon)	Ultrapure	Mono elemental	Eu	50 × 10^3^	5	20	2	2.5 × 10^2^ 5 × 10^2^ 7.5 × 10^2^ 10 × 10^2^ 12.5 × 10^2^ 15 × 10^2^ 17.5 × 10^2^	45% 60% 60% 72% 80% 90% 93%
	H-APC AC	Ultrapure	Mono elemental	Eu	50 × 10^3^	2 5 6 7	20	2	10 × 10^2^	20.0 mg/g 32.0 mg/g 47.0 mg/g 50.0 mg/g
	H-APC AC	Ultrapure	Mono elemental	Eu	50 × 10^3^	5	20	1 2	10 × 10^2^	29.0 mg/g 29.0 mg/g
	H-APC AC	Ultrapure	Mono elemental	Eu	50 × 10^3^	5	20 40 60	2	10 × 10^2^	28.9 mg/g 29.0 mg/g 29.9 mg/g
	H-APC AC	Laboratory wastewaters	Mono elemental	Eu	-	5	20	0.7	5 × 10^2^ 10 × 10^2^ 15 × 10^2^ 20 × 10^2^ 25 × 10^2^	98% 98% 99% 99% 99%
[120], 2017	AC-DETADHBA	Distilled	Multi elemental	La	5 × 10^3^	6 5 4	25	1	25 mg*	99. 6%, 145 mg/g 85% 40%
	AC-DETADHBA	Distilled	Multi elemental	La	5 × 10^3^	6	25	0.17 0.5 1	25 mg*	121 mg/g 135 mg/g 145 mg/g
	AC-COOH	Distilled	Multi elemental	La	5 × 10^3^	6	25	1	25 mg*	89.5 mg/g
[121], 2017	AC-DETADHBA	Distilled	Multi elemental	La	(10–400) ×10^3^	1–7	25	0.002,0.0083, 0.33, 0.67, 0.83	25 mg*	
[121], 2017	AC-DETADHBA	Tap water	Mono elemental	La	5 × 10^3^ 10 × 10^3^ 50 × 10^3^	6	25	1	25 mg*	99% 100% 96%
		Lake water								100% 100% 93%
		Seawater								99% 100% 93%
[121], 2017	Phosphorous functionalized nanoporous carbon	Ultrapure	Multi elemental	Nd Dy	0.5 × 10^3^	6.1 6.6	25	4	10 × 10^2^	Nd = 336 mg/g Dy = 344 mg/g
								3 0.033		Nd = 68,0% Dy = 67.0%
[122], 2017	CMK-8	Milli-Q	Multi elemental	Sm	0.02 × 10^3^	2.6	r.t.	0.5 2.5	10 × 10^2^	1 mg/g 1.5 mg/g
	CMK-8-O (CMK-8-Oxidezed)	Milli-Q	Multi elemental	Sm	0.07 × 10^3^	2.6	r.t.	0.5 1 2.5	10 × 10^2^	14 mg/g 13.8 mg/g 13 mg/g
	CMK-8-DGO (DGO: Diglycolyl-type organic)	Milli-Q	Multi elemental	La	0.0003 × 10^3^	2.6 3.8 5.7	r.t.	4	10 × 10^2^	23 mg/g 27 mg/g 22 mg/g
	CMK-8	Milli-Q	Multi elemental	Sm	(0.0025–0.025) × 10^3^	2.6	r.t.	4	10 × 10^2^	8 mg/g
	CMK-8-O (CMK-8-Oxidezed)	Milli-Q	Multi elemental	Sm	(0.05–0.2) × 10^3^	2.6	r.t.	4	10 × 10^2^	23 mg/g
	CMK-8-DGO (DGO: Diglycolyl-type organic)	Milli-Q	Multi elemental	La	(0.01–0.1) × 10^3^	2.6	r.t.	4	10 × 10^2^	10 mg/g

* There is not any mention of the volume of REEs solution used. - The optimal experimental conditions are represented by shading and the other conditions tested and described in the papers are represented on a white background (without shading).
